# Persistent Long-Term Structural, Functional, and Metabolic Changes After Stress-Induced (Takotsubo) Cardiomyopathy

**DOI:** 10.1161/CIRCULATIONAHA.117.031841

**Published:** 2018-03-05

**Authors:** Caroline Scally, Amelia Rudd, Alice Mezincescu, Heather Wilson, Janaki Srivanasan, Graham Horgan, Paul Broadhurst, David E. Newby, Anke Henning, Dana K. Dawson

**Affiliations:** 1 1 Aberdeen Cardiovascular and Diabetes Research Centre, University of Aberdeen, United Kingdom (C.S., A.R., A.M., H.W., J.S., P.B., D.K.D.).; 2 2 Department of Biomathematics and Statistics Scotland, Aberdeen, United Kingdom (G.H.).; 3 3 Department of Cardiovascular Sciences, University of Edinburgh, United Kingdom (D.E.N.).; 4 4 Department of Biomedical Imaging, University of Greifswald, Germany (A.H.).

**Keywords:** broken heart syndrome, cardiac energetics, cardiopulmonary exercise testing, stress-induced cardiomyopathy, takotsubo

## Abstract

**Background:**

Takotsubo cardiomyopathy is an increasingly recognized acute heart failure syndrome precipitated by intense emotional stress. Although there is an apparent rapid and spontaneous recovery of left ventricular ejection fraction, the long-term clinical and functional consequences of takotsubo cardiomyopathy are ill-defined.

**Methods:**

In an observational case-control study, we recruited 37 patients with prior (>12-month) takotsubo cardiomyopathy, and 37 age-, sex-, and comorbidity-matched control subjects. Patients completed the Minnesota Living with Heart Failure Questionnaire. All participants underwent detailed clinical phenotypic characterization, including serum biomarker analysis, cardiopulmonary exercise testing, echocardiography, and cardiac magnetic resonance including cardiac ^31^P-spectroscopy.

**Results:**

Participants were predominantly middle-age (64±11 years) women (97%). Although takotsubo cardiomyopathy occurred 20 (range 13–39) months before the study, the majority (88%) of patients had persisting symptoms compatible with heart failure (median of 13 [range 0–76] in the Minnesota Living with Heart Failure Questionnaire) and cardiac limitation on exercise testing (reduced peak oxygen consumption, 24±1.3 versus 31±1.3 mL/kg/min, *P*<0.001; increased VE/Vco_2_ slope, 31±1 versus 26±1, *P*=0.002). Despite normal left ventricular ejection fraction and serum biomarkers, patients with prior takotsubo cardiomyopathy had impaired cardiac deformation indices (reduced apical circumferential strain, −16±1.0 versus −23±1.5%, *P*<0.001; global longitudinal strain, −17±1 versus −20±1%, *P*=0.006), increased native T1 mapping values (1264±10 versus 1184±10 ms, *P*<0.001), and impaired cardiac energetic status (phosphocreatine/γ-adenosine triphosphate ratio, 1.3±0.1 versus 1.9±0.1, *P*<0.001).

**Conclusions:**

In contrast to previous perceptions, takotsubo cardiomyopathy has long-lasting clinical consequences, including demonstrable symptomatic and functional impairment associated with persistent subclinical cardiac dysfunction. Taken together our findings demonstrate that after takotsubo cardiomyopathy, patients develop a persistent, long-term heart failure phenotype.

**Clinical Trial Registration:**

URL: https://clinicaltrials.gov. Unique identifier: NCT02989454.

## Abstract 2

Supplemental Digital Content is available in the text.

**Clinical Perspective**

**What Is New?**

- Patients with prior takotsubo cardiomyopathy have persistent limiting symptoms and reduced exercise capacity.
- Takotsubo cardiomyopathy is associated with long-term structural and metabolic alterations in the myocardium.
- Takotsubo cardiomyopathy progresses to a persistent heart failure phenotype.

**What Are the Clinical Implications?**

- Patients should be counseled regarding the long-term symptomatic consequences of takotsubo cardiomyopathy.
- A better understanding of the mechanisms and the development of therapeutic interventions are urgently required to improve the outcome of patients with takotsubo cardiomyopathy.

Takotsubo cardiomyopathy has a dramatic clinical presentation, mimicking an acute myocardial infarction (MI).^1^ It is characterized by a precipitating major stressful event and diagnosed when invasive cardiac catheterization demonstrates unobstructed coronary arteries and ballooning of the left ventricle. This can lead to major left ventricular dysfunction and acute heart failure. In the weeks after onset, the left ventricular dysfunction gradually recovers, which leads to the commonly accepted belief that takotsubo cardiomyopathy is a transient and self-limiting condition. This concept has been reinforced by the absence of demonstrable myocardial damage on routine cardiac magnetic resonance imaging.^2^

We have previously shown severe global edema of both the left and right ventricles associated with profound cardiac energetic impairment in the acute stages of takotsubo cardiomyopathy.^3,4^ These abnormalities do not completely resolve by 4 months and occur in apparent contradistinction to the recovery of left ventricular ejection fraction. In the current study, we explored whether long-term (>1 year) abnormalities persist after an acute episode of takotsubo cardiomyopathy. Specifically, we hypothesized that a persistent impairment in cardiac energetic status (primary end point) takes place, which, despite a normalized resting left ventricular ejection fraction, results in persistent symptoms and cardiac limitation during cardiopulmonary exercise testing (peak Vo_2_ as secondary end point).

## Methods

The data-analytic methods and study materials will be made available to other researchers for purposes of reproducing the results or replicating these findings.

This was an observational cross-sectional case-control study of patients with prior (>12 months) takotsubo cardiomyopathy. All cases had to have a previously established diagnosis of takotsubo cardiomyopathy fulfilling the Mayo Clinic^5^ and the European Society of Cardiology−Heart Failure Association criteria,^6^ plus evidence of absence of fibrosis on cardiac magnetic resonance imaging. All patients had invasive coronary angiography and left ventriculography at the time of the initial diagnosis. All patients were identified from the Scottish Takotsubo Registry, and age-, sex-, and comorbidity-matched control subjects from the University of Aberdeen volunteer database were invited to participate. To match the comorbidities of the takotsubo participants precisely, control subjects were chosen to (1) be healthy and on no medication, (2) have isolated hypertension on 1 antihypertensive medication only, or (3) have diabetes mellitus (diet or metformin controlled). The study was approved by the Institutional Review Board (North of Scotland Research Ethics Committee), and all subjects gave written informed consent.

### Clinical Assessment

Patients underwent a clinical assessment for symptom burden, including New York Heart Association status and quality of life (Minnesota Living with Heart Failure Questionnaire). Control subjects were carefully assessed by an experienced clinician, incorporating a detailed history and clinical examination. All participants underwent venous blood sampling for assessment of clinical hematology and biochemistry (full blood count, urea, creatinine, and electrolytes), brain natriuretic peptide (BNP) and a range of inflammatory cytokines. Exercise tolerance and metabolic performance were assessed using treadmill cardiopulmonary exercise testing, with further measurements of BNP at peak and 15 minutes after peak exercise. Structure and function of the heart were assessed using transthoracic echocardiography and cardiac magnetic resonance, including assessment of cardiac energetic status by ^31^P-magnetic resonance spectroscopy.

### Biomarkers of Cardiovascular Homeostasis and Inflammatory Status

Serum was separated after centrifugation at 50 g for 10 minutes and stored at −80°C. BNP concentrations were determined using an immunoassay (Alere Triage MeterPro). Quantification of serum cytokine concentrations—growth-regulated protein or chemokine (C-X-C motif) ligand 1, tumor necrosis factor-α, interferon γ, monocyte chemoattractant protein 1, and the interleukins (IL-1, IL-6, IL-8 (chemokine ligand 8), IL-10, IL-12p40)—were performed using a bespoke commercially available kit (MILLIPLEX MAP Human Cytokine/Chemokine Magnetic Bead Panel; Merck Millipore).

### Cardiopulmonary Exercise Testing

Cardiopulmonary exercise testing was performed on a treadmill (Marquette 12000, GE) integrated with a metabolic system (Quark PFT, Cosmed) using a progressive incremental ramp protocol of 1 km/h and base elevation of 1% gradient every minute until volitional exhaustion. Oxygen consumption (Vo_2_) was measured breath by breath, and 12-lead ECG, heart and respiratory rate, blood pressure, and oxygen saturations were measured throughout. Any test with a respiratory exchange ratio (RER) <1.1 was classed as suboptimal, and subjects with a forced expiratory volume end of the first second <70% of predicted were excluded from these analyses.

### Transthoracic Echocardiography

Echocardiography was performed using a Vivid E9 equipped with a 2.5-MHz (M5S) transducer (GE Vingmed) by a single experienced British Society of Echocardiography-accredited sonographer. Three cardiac cycles in each of the standard parasternal long-axis, short-axis, apical 4-, apical 3-, and apical 2-chamber views were obtained at end-expiratory breath hold at a frame rate of ≥85 Hz and stored for off-line analysis. Any subject with left bundle-branch block on ECG was excluded from the strain and deformation analysis. Image analysis was performed using EchoPAC software (version 1.13) as previously described measuring left ventricular longitudinal, radial and circumferential strain, and deformation indices.^7^

### Cardiac Magnetic Resonance Imaging and ^31^P-Magnetic Resonance Spectroscopy

All participants were scanned on a 3T Philips Achieva scanner (Best). ^31^P-CMR spectroscopy (^31^P-CMRS) was acquired using a 14-cm diameter transmit and receive ^31^P surface coil as described previously.^3^ A nonwater-suppressed ^1^ H point-resolved spectroscopy acquisition was used to monitor resonance frequency determination and B_0_ shimming over the ^31^P-CMRS volume of interest, which was positioned to cover the entire interventricular septum. The ^31^P-CMRS acquisition was an ECG-gated image-selected in vivo spectroscopy sequence, triggered to mid-late diastole, with a repetition time of ≥10 seconds. A 5-channel cardiac coil was used to acquire cine imaging, early and late gadolinium enhancement (Gadovist, 0.1 mmol/kg) with swap of the phase-encoding direction, and pre- and postcontrast Modified Look-Locker Imaging T1 mapping.^8^ Data for T1 mapping were acquired with 3(3)3(3)5 and 5(3)3 schemes for native and postcontrast T1 measurement, respectively. ^31^P-CMRS data were analyzed in JMRUi3.0 as described previously.^3^ The phosphocreatine/γ-adenosine triphosphate (PCr/γATP) ratio (which is the gold standard for in vivo assessment of myocardial energetic status)^9^ was determined after the γATP was corrected for blood contamination and PCr/γATP ratios were saturation-corrected as described previously.^10–12^ To ensure that spectra were of good quality, Cramér-Rao standard deviations of all peaks were calculated, and only those <20% were accepted. The CMR images were analyzed in CMR Tools (Cardiovascular Imaging Solutions) for computation of left ventricular volumes and mass. T1 maps were imported into Segment (Medviso), and T1 values were generated for each of the 16 segments of the 17-segment model^13^ (omitting the true apex), and the myocardial extracellular volume fraction was calculated as described previously.^4^

Our interobserver variabilities for strain echocardiography, ^31^P-CMRS, and CMR have been reported previously and ranged between 3 and 6±1% to 2% for all strain echocardiography parameters, 1.5 and 2.7±0.5 to 1.5% for cardiac magnetic resonance inclusive of T1 mapping, and 5±2% for cardiac spectroscopy. Our interstudy variability for healthy volunteers were 5±2% for strain echocardiography, 2±0.5% for cardiac magnetic resonance and T1 mapping, and 6±2% for PCr/γATP ratio.^3,4,7^

### Statistical Analysis

Statistical analysis was performed using IBM SPSS statistics version 24.0 and R version 3.2 (R Foundation for Statistical Computing). Case and control groups were compared by analysis of variance, with age and sex as covariates, to reduce the unexplained residual variability and increase the power of the comparison. Because some medications/comorbidities were not present in control subjects, data were analyzed both with and without these as covariates (the data without these additional covariates are reported because they did not affect the conclusions). Log-transformed values were used where distributions appeared skewed. Means were calculated and adjusted for age and sex distribution in the whole dataset. Data are shown as mean±SEM or median (range). Statistical significance was set at *P*<0.01 rather than the usual 0.05 to reduce the risk of type I errors resulting from multiple hypothesis tests.

## Results

Thirty-seven patients with prior takotsubo cardiomyopathy were recruited at a median time of 20 months (range 13–39) from the index event. They were predominantly middle-age or elderly (median 65 years, range 34–81) women. Their demographics and acute takotsubo episode details are summarized in Table 1. Thirty-seven control subjects of a similar median age 64 (range 34–86), sex (36 female), and comorbidity distribution were enrolled (Table 1). The diagnosis of a psychiatric illness predated the acute takotsubo event in all cases.

**Table 1.**
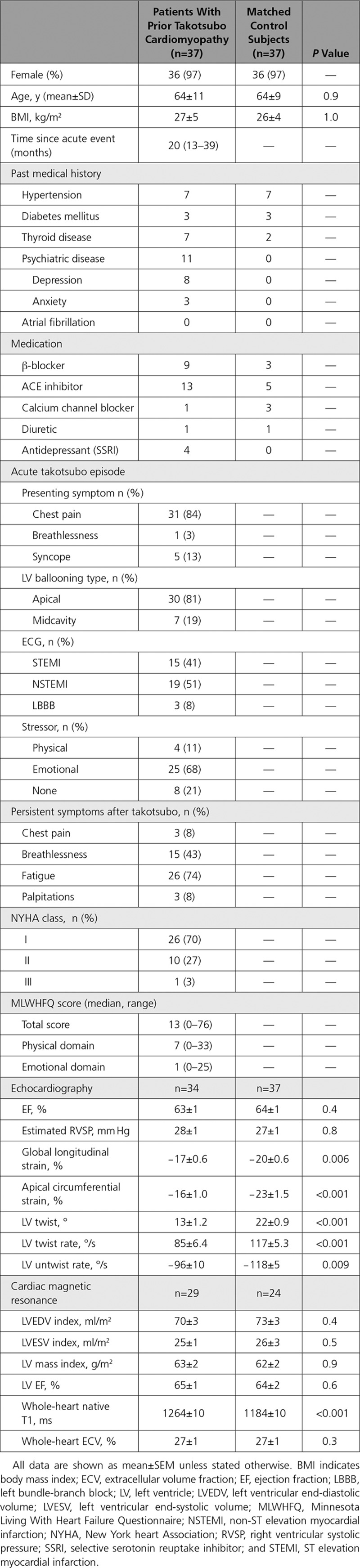
Characteristics of Study Population

### Symptoms

The majority (88%) of patients with prior takotsubo cardiomyopathy had symptoms including fatigue (74%), shortness of breath (43%), palpitations (8%), and chest pain (8%). Most patients were New York Heart Association class I (70%), 10 patients were New York Heart Association class II (27%), and 1 patient was New York Heart Association class III (3%). All control subjects were asymptomatic.

**Figure 1.**
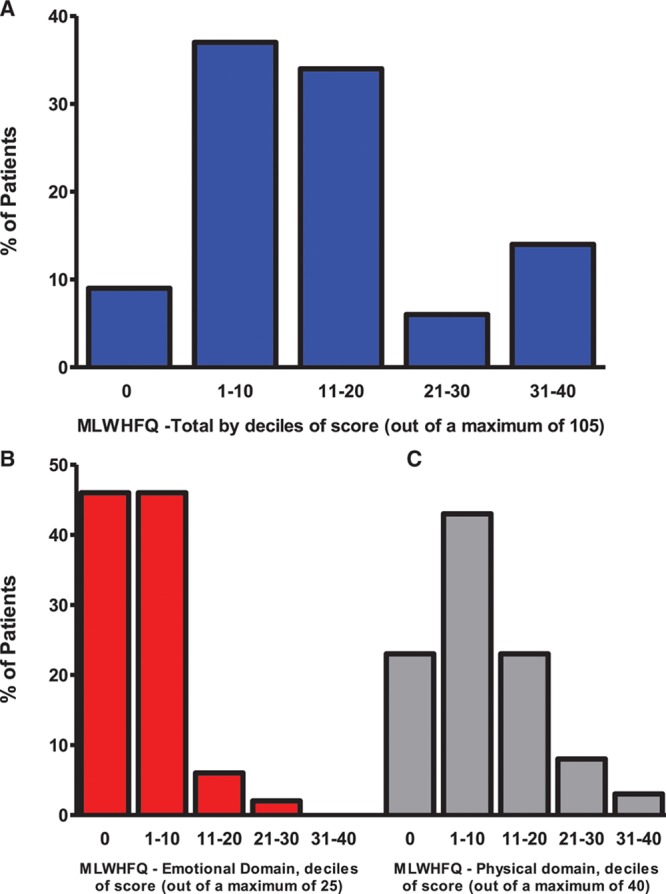
**Minnesota Living with Heart Failure Questionnaire (MLWHFQ) scores by decile. A**, MLWHFQ total score. **B**, Emotional domain. **C**, Physical domain.

Thirty-five patients completed the Minnesota Living with Heart Failure Questionnaire (Figure 1). The median total score was 13 (range 0–76). Only 3 patients reported a score of 0, and patients scored higher in the physical domain with a median of 7 (range 0–33) than in the emotional domain with a median of 1 (range 0–25).

### Exercise Capacity (Peak Oxygen Consumption)

**Figure 2.**
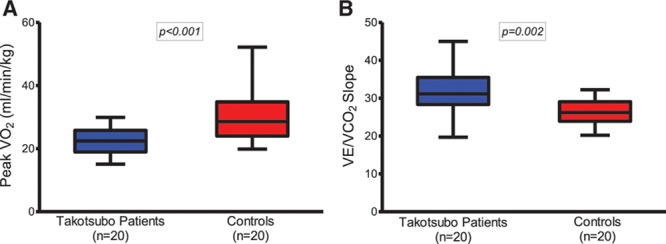
**Cardiopulmonary exercise data in patients with takotsubo cardiomyopathy and matched control subjects. A**, Peak Vo_2_. **B**, VE/Vco_2_ slope. Data shown as median, 25th, and 75th percentiles and maximum and minimum (whiskers).

Of the 37 patients with prior takotsubo cardiomyopathy, 1 had both breathlessness and ischemic changes on the ECG during exercise and stopped before an RER of 1.1. Only 20 (54%) patients were able to exercise to an RER of 1.1, and their results are shown in Table 2, Figure 2, and Table I in the online-only Data Supplement. No desaturation on exercise occurred in either group. Blood pressure and heart rate responses as well as the breathing reserve were similar between groups. Compared with control subjects, patients with prior takotsubo cardiomyopathy had a lower peak Vo_2_ and a higher VE/Vco_2_ slope (*P*<0.001 and *P*=0.002, respectively). These findings were consistent irrespective of the exclusion of those subjects receiving β-blocker therapy (n=5 patients with takotsubo cardiomyopathy; *P*=0.004 for peak Vo_2_ and *P*=0.003 for VE/Vco_2_ slope). Patients with prior takotsubo cardiomyopathy had a lower metabolic equivalent than the control subjects (*P*=0.001). There was no significant difference between groups in the BNP concentrations at any of the time points assessed (rest, peak exercise, or 15 minutes after peak exercise) (Table 2).

**Table 2.**
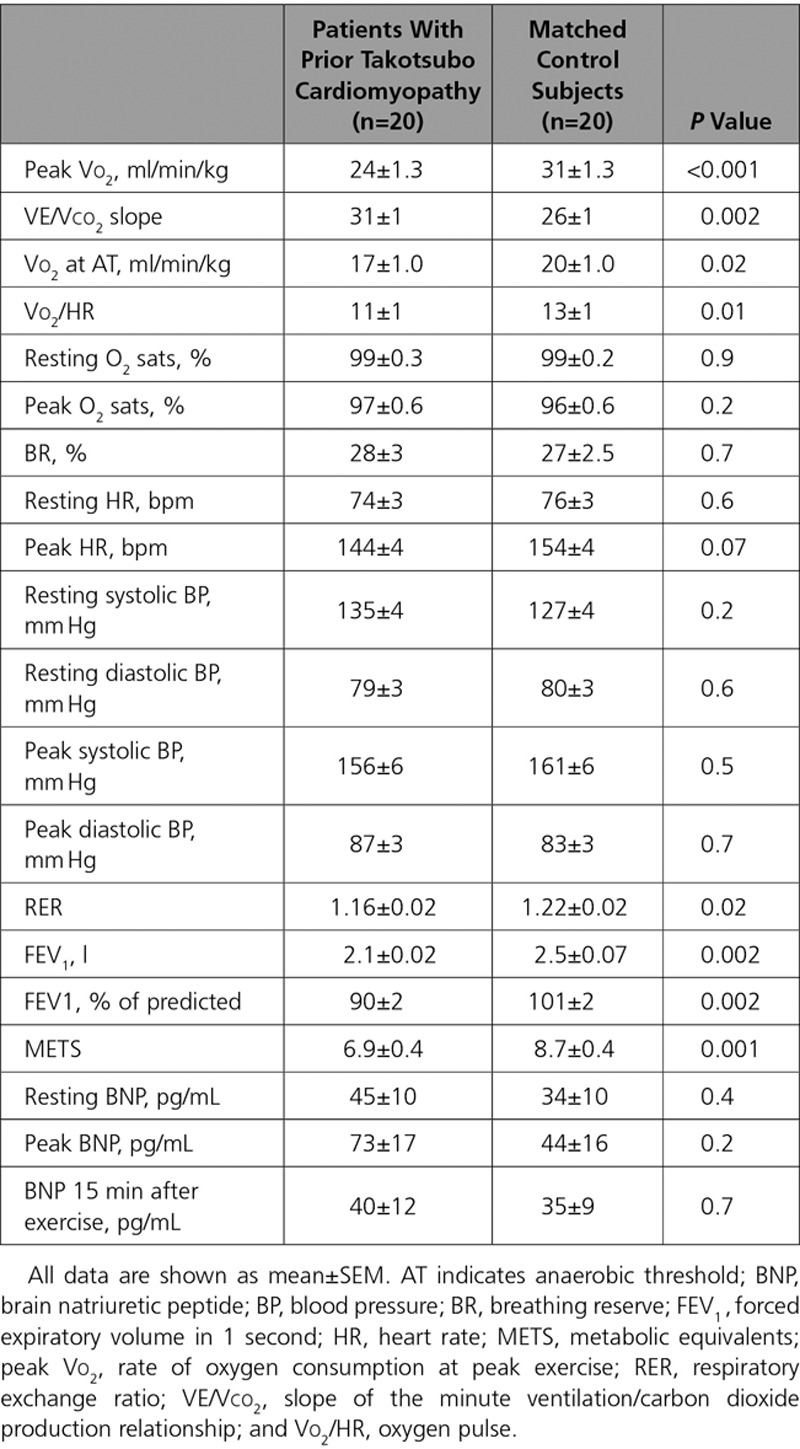
Cardiopulmonary Exercise Testing in Patients With Prior Takotsubo Cardiomyopathy and Matched Control Subjects

### Echocardiography

Left ventricular ejection fraction and estimated pulmonary artery pressure were similar in patients with prior takotsubo cardiomyopathy and control subjects (Table 1). However, compared with control subjects, the global left ventricular longitudinal and apical circumferential strain were reduced (*P*=0.006 and *P*<0.001, respectively). As a result, global left ventricular twist, twist rate, and untwist rate were reduced in patients with prior takotsubo cardiomyopathy (*P*<0.001, *P*<0.001, and *P*=0.009, respectively) (Table 1 and Table II in the online-only Data Supplement).

### Cardiac Magnetic Resonance

Indexed left ventricular volumes, mass, and ejection fraction were similar in patients with prior takotsubo cardiomyopathy and control subjects (Table 1). None of the participants had evidence of late gadolinium enhancement. However, whole heart native T1 values were higher in patients with prior takotsubo cardiomyopathy than control subjects (*P*<0.001). The differences were seen in the basal, midcavity, and apical segments (Table III in the online-only Data Supplement). No difference was found in the whole heart-calculated extracellular volume fraction (*P*=0.3).

**Figure 3.**
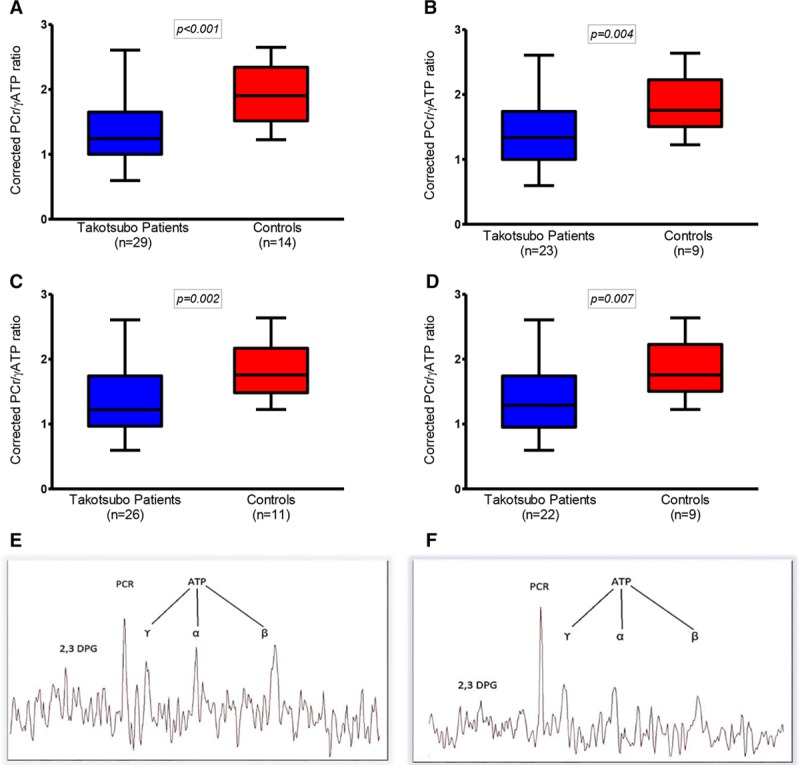
**Corrected phosphocreatine (PCr)/γ-adenosine triphosphate (γATP) ratio in patients with takotsubo cardiomyopathy and matched control subjects. A**, All subjects. **B**, Excluding subjects with hypertension. **C**, Excluding subjects with diabetes mellitus. **D**, Excluding subjects with both hypertension and diabetes mellitus. Data shown as median, 25th, and 75th percentiles and maximum and minimum (whiskers). Representative spectral analyses. **E**, Patient with previous takotsubo cardiomyopathy. **F**, A healthy control. The takotsubo patient shows a reduction in PCr/γATP ratio.

Resting cardiac energetic status (PCr/γATP ratio) and cardiac spectra were markedly different in patients with prior takotsubo cardiomyopathy (Figure 3). When the subject groups were divided by comorbidity status (hypertension, diabetes mellitus), they had similar PCr/γATP ratios as those without (Table 3, Figure 3).

**Table 3.**
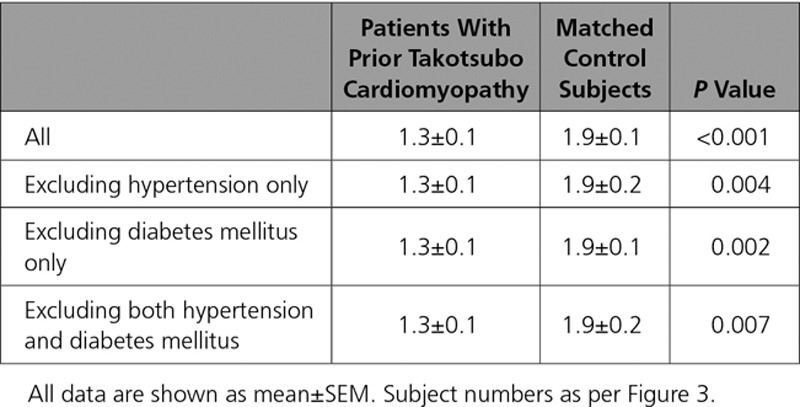
Corrected Phosphocreatine (PCr)/γ-Adenosine Triphosphate (γATP) Ratio in Patients With Prior Takotsubo Cardiomyopathy and Matched Control Subjects

### Serum Cytokine Profiles

An apparent trend appeared for higher serum growth-regulated protein or chemokine ligand 1concentrations in patients with prior takotsubo cardiomyopathy (*P*=0.08), but all other cytokine concentrations were similar between the groups (Table IV in the online-only Data Supplement).

Clinical hematology and biochemistry were within normal limits in all participants (data not shown).

## Discussion

This is the first detailed cross-sectional case-control study investigating the long-term structural and functional consequences of takotsubo cardiomyopathy. We demonstrate that most patients with prior takotsubo cardiomyopathy have persistent symptoms of heart failure because of physical rather than emotional disability. This finding is consistent with their poor exercise tolerance and evidenced by objective reductions in peak Vo_2_ because of cardiac limitation on cardiopulmonary exercise testing (increased VE/Vco_2_ slope). In turn, these factors appear to be related to alterations in native T1 values, impaired left ventricular strain patterns, and reduced cardiac energetic status. Taken together, these findings demonstrate a substantial and persistent adverse alteration in long-term cardiac morbidity associated with takotsubo cardiomyopathy and define a new clinical phenotype in this population.

Our study has a number of important strengths. First, we used a rigorous definition of takotsubo cardiomyopathy and specifically excluded patients with any prior overt or covert cardiac disease. Second, we had a robust sample size and specifically incorporated a control population that was well matched for important characteristics and typical comorbidities. Third, we deliberately studied patients remote from their index event to ensure that we were exploring effects that could not be attributable to the acute index episode. Finally, we undertook highly detailed symptomatic and objective assessments of cardiac structure and function using state-of-the-art techniques. Therefore, we believe our findings are robust, informative, and novel.

### Study Rationale

Despite its dramatic clinical presentation, a fundamental characteristic of takotsubo cardiomyopathy is the spontaneous recovery of the left ventricular ejection fraction, which returns to normal or near normal in all patients over a variable period of time (days to weeks). In contrast to this assumption of rapid and complete recovery, 2 large registries have recently and independently reported that the long-term mortality of takotsubo cardiomyopathy appears comparable to that of patients with MI.^14–16^ Because these prognostic registry data are in apparent contradiction with the universally observed resolution of left ventricular ejection fraction, the key question arising is: what additional processes intervene to confer on these patients such an unexpectedly poor prognosis? Our study provides important mechanistic data that may help explain some of these findings.

### Exercise Capacity and Metabolic Performance

It was surprising and noteworthy that nearly half of our patients with prior takotsubo cardiomyopathy were unable to exercise sufficiently to achieve the required RER of 1.1. Their attempted but unachieved cardiopulmonary exercise test strengthens the argument that these patients experience marked exercise limitation, mostly represented by their symptoms of general fatigue. Even in those patients who achieved an RER >1.1, several important abnormal characteristics were present, including lower peak Vo_2_ and higher VE/Vco_2_ slope. Unlike other typical heart failure phenotypes, patients with prior takotsubo cardiomyopathy had an appropriate blood pressure response and no chronotropic incompetence with exercise. Their normal oxygen saturation at maximal exercise and normal breathing reserve ruled out a respiratory cause for their peak Vo_2_ reduction. Despite their normal left ventricular ejection fraction, these metabolic characteristics are strongly suggestive of a heart failure phenotype. Both peak Vo_2_ and VE/Vco_2_ slope are predictors of cardiovascular morbidity and mortality in heart failure of several etiologies.^17–19^ The finding of a lower forced expiratory volume in patients with prior takotsubo cardiomyopathy is perhaps surprising and unexplained, although patients with other heart failure syndromes also have a reduced forced expiratory capacity.^20^ Although only detected as a mild reduction (all forced expiratory volumes were >90% of predicted), this may also be a feature of post-takotsubo–cardiomyopathy, although the less likely possibility of subclinical and previously unrecognized lung pathology of a different etiology in this group has not been entirely ruled out.

### Functional and Structural Abnormalities of the Left Ventricle and Cardiac Energetic Status

How do we account for the increased symptoms and markedly reduced exercise capacity in the face of an apparently normal left ventricular ejection fraction? We report here several important abnormalities of left ventricular performance. Echocardiography strain deformation data reveal subtle abnormalities located mostly in the left ventricular apex. Together with the significantly basal, midcavity, and apical prolongation of T1 values suggesting microscopic fibrosis,^21,22^ this finding points to the fact that distinctive and regional intramyocardial remodeling processes persist after an acute episode of takotsubo cardiomyopathy, suggesting that the heart loses some if its wringing motion mostly in the regions acutely affected (the apex and midcavity in this cohort studied in this report). Although these changes are subtle and do not result in any marked, readily identifiable remodeling, such as that seen to occur after MI, they do have an impact on the functional status and development of heart failure symptoms. These data are in keeping with the prediction of the acute invasive hemodynamic studies performed by Medeiros et al, ^23^ who predicted that if the mismatch observed in the acute phase in the ventricular-arterial coupling of these patients remains prolonged, it could conceivably contribute to chronic myocardial remodeling. Finally, further evidence comes from the reduction in cardiac energetic status of these patients, which is in keeping with their exercise/metabolic testing. The lower PCr/γATP ratio in patients with takotsubo suggests that this abnormality, once established, is ongoing and likely contributory to the chronic takotsubo phenotype, at least in part. The abnormal cardiac energetic status did not appear to be driven by other pathologies such as hypertension or diabetes mellitus, which are known to negatively impact on cardiac energetic status.^24–26^ These comorbidities do so in more advanced clinical stages compared with our patients with takotsubo (no patient in our cohort was on >1 antihypertensive or 1 antidiabetic medication, and comorbidities were well controlled on this regime without any evidence of end-organ damage).

### Biomarkers of Cardiovascular Homeostasis and Inflammation

Given the remarkable intensity and extent of edema present in the myocardium in the acute stages of takotsubo cardiomyopathy, which was shown to be slow to resolve, we thought it important to explore the long-term pro- and anti-inflammatory status of these patients. Although the mean concentrations of all proinflammatory cytokine concentrations were numerically higher in patients with prior takotsubo cardiomyopathy, no statistically significant differences were identified. We therefore cannot exclude a low-grade systemic or myocardial inflammatory state, and this warrants further investigation in a larger sample size. Similarly, although the BNP concentrations were not significantly different from the control subjects, they were above accepted resting reference values for normalcy.^27^ The above-normal BNP values may represent increased myocardial stretch because of increased filling pressures, a persistent low-grade inflammation status, or both. Further studies are required to elucidate this. However, this study was not powered to assess any biomarker end points, so these data are of hypothesis-generating value at this stage.

### Study Limitations

We acknowledge our study’s weaknesses. From our study design, it is not possible to determine whether these marked abnormalities described here are a consequence of takotsubo cardiomyopathy or predate and predispose the patient to develop this condition. Further work is needed to establish whether predisposing factors are identifiable so that susceptible individuals can be investigated or offered potential preventive therapies. Moreover, it would be important to establish cause and effect as well as potential mechanisms of pathways of this serious condition. We also acknowledge the potential for case selection bias, in that symptomatic patients may have been more inclined to volunteer for inclusion in our study. Future prospective cohorts may be able to address this concern.

### A Gap in Knowledge

Overall, the data in this study fill an important gap of currently missing but much needed information for the appropriate clinical care of patients with takotsubo. Many descriptive studies have focused on the acute presentation of takotsubo cardiomyopathy. The invariable recovery of left ventricular ejection fraction has misled the medical community into believing that takotsubo cardiomyopathy is transient and self-limiting. Despite a well-recognized early mortality^28–30^ and establishment of clinical features pointing to those at risk,^31,32^ no data have described the physiological and clinical status of these patients long term. To the best of our knowledge, these data, which demonstrate that patients with takotsubo have symptoms and objective findings compatible with a heart failure syndrome, are unique and novel in the takotsubo literature. These findings should impact clinical practice primarily in the counseling and follow-up of such patients, and they should encourage further research into therapies to mitigate symptoms and improve prognosis.

## Conclusions

We describe a new clinical phenotype in patients who suffered an acute takotsubo episode >1 year previously. This is characterized by impaired cardiac energetic status and reduced maximal oxygen consumption on exercise because of significant cardiac limitation. Our findings demonstrate that patients with prior takotsubo cardiomyopathy develop a heart failure phenotype with a significant impact on quality of life.

## Sources of Funding

The HEROIC study was funded by the British Heart Foundation Project Grant no. PG/15/108/31928 (D.K.D.), the Josephine Lansdell British Medical Association 2015 Award (D.K.D.), and the Chief Scientist Office CGA-16-4 Catalytic Grant (D.K.D). D.E.N. is supported by the British Heart Foundation (CH/09/002) and a Wellcome Trust Senior Investigator Award (WT103782AIA).

## Disclosures

Dr Dawson has a research agreement with Philips Healthcare and holds an MTA with AMAG Pharmaceuticals. The other authors declare no conflicts of interest.

## Supplementary Material
