# Supplementary material for: Persistent Long-Term Structural, Functional, and Metabolic Changes After Stress-Induced (Takotsubo) Cardiomyopathy
Source: Circulation. 2018 Mar 5;137(10):1039–48. doi: 10.1161/CIRCULATIONAHA.117.031841 (PMC5841855; doi:10.1161/CIRCULATIONAHA.117.031841)

Supplemental Material

Longitudinal assessment

Structural, functional and metabolic imaging assessment were available in a subgroup of patients (n=13) who enrolled in our prior studies.[3,4] While the left ventricular ejection fraction recovered promptly at 4 months and remains unchanged thereafter, the wall motion score index showed a further small recovery over time (**Supplemental Figure**). The severely depressed cardiac energetics in the acute phase had a modest improvement at 4 months but this was not sustained in the longer term. Increased T1 values also partly subsided at 4 months (representing a reduction in myocardial edema) but became elevated again in long term in keeping with the similar pattern observed for left ventricular mechanics (and implying that a different remodelling process takes place in the longer term, most likely microscopic fibrosis (**Supplemental Figure**).

Supplemental Table 1.

Cardiopulmonary exercise data in patients with prior takotsubo cardiomyopathy and matched control subjects.

	**Patients with prior takotsubo cardiomyopathy (n=20)**	**Matched control subjects (n=20)**	**p-value**
VE, l/min	57±3	73±3	**<0.001**
MVV, l/min	86±3	100±3	**0.004**
Respiration rate, breaths/min	36±1	40±1	0.1
Tidal Volume, l	1.6±0.05	1.8±0.05	**0.009**

VE=Ventilatory equivalent, MVV = maximum voluntary ventilation.

All data shown as mean±SEM

Supplemental Table 2. Echocardiography data in patients with prior takotsubo cardiomyopathy and matched control subjects.

	**Patients with prior takotsubo cardiomyopathy n=34**	**Matched control subjects n=37**	**p-value**
LVEF, %	63±1	64±1	0.5
TR max, m/s	2.3±0.1	2.4±0.1	0.4
E, m/s	0.75±0.04	0.74±0.04	0.9
A, m/s	0.84±0.03	0.77±0.03	0.1
E/A	1.0 ±0.6	1.0±0.8	0.9
TAPSE	2.3±0.1	2.5±0.1	0.04
Basal radial strain,%	37.3±2.6	36.3±2.5	0.8
Basal circumferential strain, %	-14.4±1.1	-15.2±1.0	0.6
Mid-cavity radial strain, %	40.9±3.1	47.3±3.1	0.2
Mid-cavity circumferential strain, %	-15.5±1	-15.9±1	0.7
Apical radial strain, %	28.9±2.8	24.1±2.6	0.2
Apical rotation, °	7.2±1.6	10.4±1.3	0.1
Basal rotation, °	-5.2±1.6	-6.7±1.3	0.5

LVEF= LV Ejection fraction; LVESV=LV end systolic volume; LVEDV=LV end diastolic volume; TR max= maximum velocity of Tricuspid regurgitation; RVSP=Right ventricular systolic pressure; E=early ventricular filling velocity; A=late ventricular filling velocity; E/A = ratio of early to late ventricular filling velocity; TAPSE = Tricuspid annular plane systolic excursion.

All data shown as mean±SEM.

Supplemental Table 3.

T1 mapping data in patients with prior takotsubo cardiomyopathy and matched control subjects.

	**Patients with prior takotsubo cardiomyopathy (n=28)**	**Matched control subjects (n=24)**	**p value**
**Native T1, ms**			
Basal	1259±7	1224±8	**0.002**
Mid-cavity	1249±8	1189±9	**<0.001**
Apical	1296±18	1168±19	**<0.001**
**ECV, %**			
Basal	27± 1	29±1.0	0.3
Mid-cavity	25± 1	27±1	0.1
Apical	27±1	29± 1	0.3

ECV = Extracellular Volume;

All data shown as mean ± SEM

Supplemental Table 4:

Serum cytokines concentrations in patients with prior takotsubo cardiomyopathy and matched control subjects.

Cytokines	Patients with prior takotsubo cardiomyopathy (n=29)	Matched control subjects (n=18)	p-value
IL-1, pg/ml	6.4±3.4	0.9±0.2	0.2
IL-6, pg/ml	23±7	18±10	0.7
IL-8 (CXCL8), pg/ml	48±11	24±14	0.2
IL-10, pg/ml	24±7	19±7	0.6
IL-12p40, pg/ml	56±18	53±19	0.8
MCP-1, pg/ml	792±60	697±75	0.3
GRO (CXCL1), pg/ml	2017±308	1910±384	0.08
TNF-α, pg/ml	19±2	17±2	0.5
IFN – γ, pg/ml	64±19	52±19	0.6

Interleukins (IL-6, IL-8(CXCL8), IL-10, IL-12p40); MCP-1= Monocyte chemoattractant protein 1; GRO = growth regulated protein or chemokine (C-X-C motif) ligand 1; TNF-α=tumour necrosis factor alpha; IFN-γ=interferon gamma

All data shown as mean±SEM

Supplemental Figure: Time course of cardiac changes after takotsubo cardiomyopathy. Left ventricular ejection fraction (LVEF) and wall motion score index (a), corrected phosphocreatine (PCr)/γ-adenosine triphosphate (ATP) ratio (b), T1 mapping (c), Global longitudinal strain (d), left ventricular twist (e) and left ventricular untwist rate (f) in a subgroup of patients seen longitudinally at 3 time-points. Data shown as mean±SD (a) or median, 25th and 75th centiles and maximum and minimum (whiskers) (b-f)

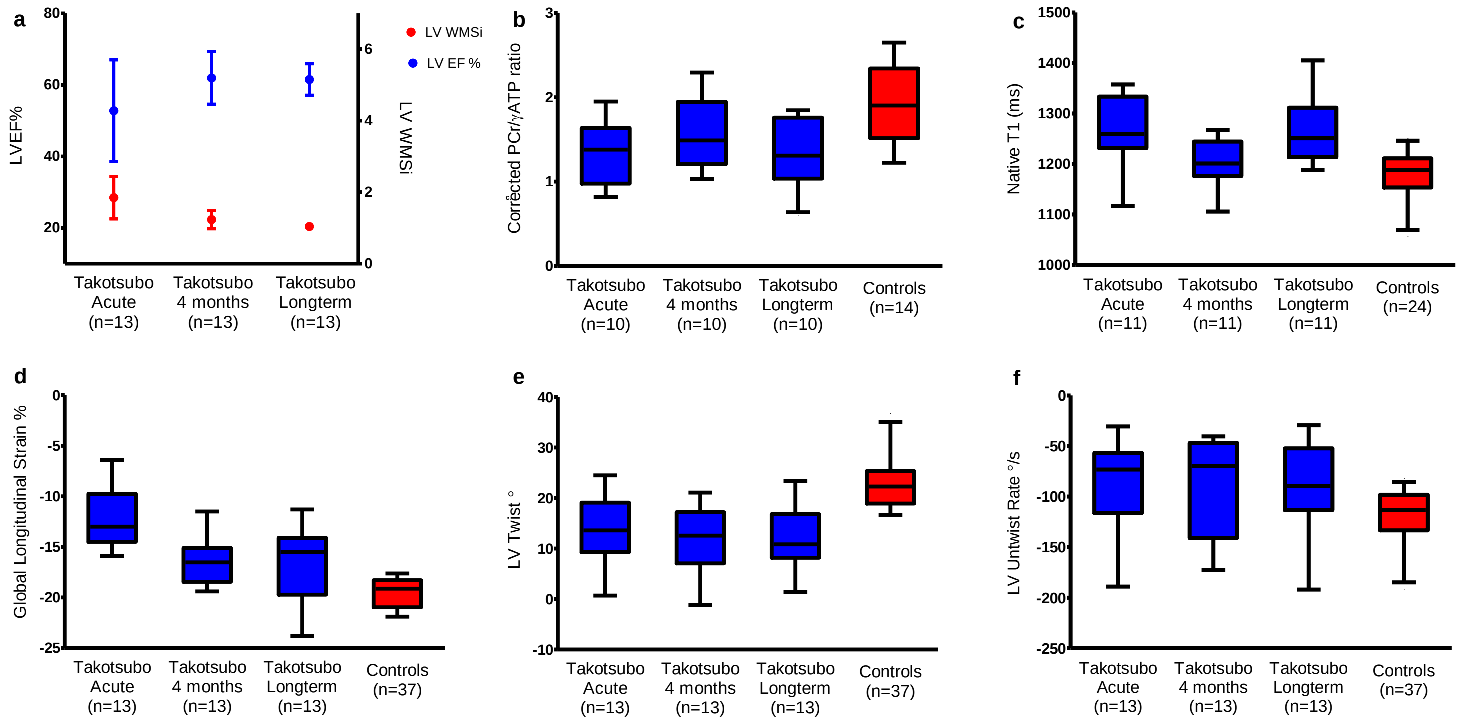

Supplement: Supplementary file 1 [file cir-137-1039-s001.pdf]
